# Comparison of the Effects of Pre-Anesthetic Administration of Normal Saline, Ringer and Voluven on the Spread of Sensory Block With Hyperbaric Bupivacaine Spinal Anesthesia

**DOI:** 10.5812/aapm.17939

**Published:** 2014-05-12

**Authors:** Elham Memary, Alireza Mirkheshti, Morteza Jabbari Moghaddam, Dariush Abtahi, Mehdi Yaseri, Farnaz Kamali

**Affiliations:** 1 Anesthesiology Research Center, Shahid Beheshti University of Medical Sciences, Tehran, Iran; 2 Department of Epidemiology and Biostatistics, Tehran University of Medical Sciences, Tehran, Iran

**Keywords:** Anesthesia, Spinal, HES 130-0.4, Hydroxyethyl Starch Derivatives

## Abstract

**Background:**

Spinal anesthesia is an important and commonly used method for surgical anesthetic in operating rooms. However, even with identical drug dosage and administration mode, the extent of drug distribution in vivo is highly variable and difficult to control. Preanesthetic administration of fluids immediately before spinal anesthesia (preload) is normal practice. The choice of fluid type may affect drug distribution as well as the duration and level of the block.

**Objectives:**

We examined whether preloads of normal saline, Ringer, or hydroxyethyl starch has different effects on the time it takes to reach maximum block, and the distribution and duration of spinal block level.

**Patients and Methods:**

This was a randomized trial and the 150 patients selected were evenly divided into three groups and given; normal saline, Ringer, or hydroxyethyl starch 130/0.4f luids. Preload was given at 10 mL/kg for the normal saline and Ringer groups, and 5 mL/kg for the hydroxyethyl starch group, 10 min before the spinal anesthesia. Sensory block levels were recorded every 5 min until 30 min after spinal anesthesia and then at 60 and 90 min. Time taken to reach maximum and median sensory block, maximum and median level of block, duration of block, and hemodynamic status were recorded.

**Results:**

There were no statistically significant differences in the demographic characteristics between the three groups. Maximum block was higher in normal saline compared to Ringer (P = 0.029). Time taken to reach maximum block was greater in Ringer compared to both normal saline (P = 0.001) and hydroxyethyl starch (P = 0.003). Normal saline had a longer duration of sensory block T10 compared to Ringer and hydroxyethyl starch (P = 0.03).

**Conclusions:**

Preload fluids have an impact on the level, distribution and duration of sensory block in spinal block. Of the three fluids, normal saline produced the greatest maximum and longest duration of block, whereas time taken to reach maximum block was longer in the Ringer group.

## 1. Background

Spinal anesthesia is one of the oldest anesthesia methods. Despite the development of a number of safe and more sophisticated techniques of general anesthesia, spinal anesthesia has gained in popularity because of its efficacy, simplicity, safety, introduction of new drugs with fewer side effects, and more benefits for certain patient populations and surgical procedures (1). The greatest challenge of the technique is to control the spread of the local anesthetic through the cerebrospinal ﬂuid (CSF), in order to provide a block that is adequate (in both extent and degree) for the proposed surgery, yet without producing unnecessary spread which increases the risk of complications (2, 3).

Since an insufficient level and duration of block can lead to pain felt during the operation, it is necessary to estimate the level and duration of sensory and motor block, and the time it will take to achieve the desired block level. If these parameters are not as expected, cessation of the operation or a change to general anesthesia may be indicated. This change is sometimes not possible, or at least very difficult, because of the patient’s condition and intra-operative parameters. On the other hand, an unintentional increase in the level of anesthesia or duration of block can increase hemodynamic complications, duration of recovery, and delay both the onset of ambulation and discharge (4).

From the time when spinal anesthesia was first used, much effort has been made to determine which factors affect anesthesia efficacy. Many clinicians have recommended the rapid administration of fluids before spinal anesthesia (prehydration) to reduce complications, but the effects of these fluids remain controversial (5).

Previous studies have focused on the amount and type of fluids, or they have compared fluids with vasopressors to reduce complications (6-8). Studies that have focused on the effect of the anesthetic solution's baricity on the extent of the block, have shown that if the spinal anesthetic is injection into the cerebrospinal fluid (CSF), and the patient’s position remains unchanged for at least five minutes, these drugs will preferentially distribute to the lower or dependent parts (9, 10). Conversely, hypobaric solutions mainly distribute to the independent parts, for example, in a sitting position, the direction of hypobaric solution distribution would be cephalad (11).

CSF volume is the most important factor which affects the level of spinal anesthesia. To determine the level and duration of a sensory block it is important to understand what factors affect the CSF volume and how the volume affects the efficacy of drugs with different baricities. Recent studies have highlighted that the pre-load solution type may affect CSF volume (1, 12).

## 2. Objectives

We decided to investigate the effects of different types of solutions on the efficacy of the anesthetic. We chose to measure efficacy by measuring the time taken to reach maximum and median sensory block, level of block, and duration of block. In order not to confound the results, we limited the study to patients receiving just one type of spinal anesthesia. 

Our main aim was to compare the effects of an intravenous infusion of; normal saline (NS), Ringer (R), and hydroxyethyl starch (HES) solutions, administered before a spinal anesthesia with hyperbaric bupivacaine on the sensory block in patients who were candidates for lower limb elective surgery.

## 3. Patients and Methods

Patients scheduled to undergo elective lower extremity surgery with spinal anesthesia in the Imam Hossein Hospital for less than three hours were eligible. Inclusion criteria were: aged 18-70 years, ASA I or II, spinal cord length between 66 and 76 cm (male) or 56 and 66 cm (female). The spinal cord length was measured by a designated staff member using the C7 spinous process to the sacral hiatus (in a sitting position, face forward and flat legs in rest on the operating table).

Exclusion criteria were: ASA III, and IV, cardiac-respiratory-renal insufficiency or underlying hepatic failure, inadequate linguistic contact, and inability to cooperate in order to determine the block level, smoker or opium user, spinal diseases or vertebral deformities, spinal column length out of the defined range, neurologic deficits or coagulopathies and contraindications for spinal anesthesia. No premedication was given before surgery. 

Routine monitoring and primary records of the vital signs were performed. Patients were randomly allocated to receive one of three fluid types: normal saline (NS); Ringer (R) (Samen, Iran); or hydroxyethyl starch 130/0.4 (HES) (trade name Voluven, Frezinus, Germany). 

All patients fasted for eight hours before surgery. Fluids were infused for 10-15 min in the operating room's waiting area prior to spinal anesthesia by an anesthesiologist. Volumes infused were 10 mL/kg for NS and R, and 5 mL/kg for HES. Baseline values of heart rate, noninvasive arterial blood pressure, and O_2_ saturation were recorded on arrival in the operating room.

A second anesthesiologist, blinded to the preload fluid type administered, performed the spinal anesthesia at the third lumbar interspace (L3– 4), using the midline approach, with the patients in the right lateral decubitus position, and assessed the sensory and motor levels. Local anesthesia was 2 mL of lidocaine 2% with a 25-gauge needle, and spinal anesthesia was performed via a 25-gauge Whitacre spinal needle (BD Whitacre needle, BD Medical System, New Jersey, USA), with 3 mL (15 mg) hyperbaric bupivacaine at 0.2 mL/sec, after ensuring the correct needle position and CSF drainage. 

Immediately after anesthesia, the patient was turned to the supine position where they remained for 15 min. The patients were given oxygen through nasal prongs at 3-5 L/min. The chart of sensory and motor block level and hemodynamic status was completed.

The median sensory nerve block levels were evaluated by the pinprick test in the midline region of the skin dermatomes using a 25-gauge Whitacre needle, and recorded according to the relevant dermatome. The sensory block levels were recorded every 5 min until 30 min after spinal anesthesia and then at 60 and 90 min. We recorded the maximum sensory block reached at each time point, the peak sensory block reached, the time required to achieve sensory block in T10, and the duration of sensory block at T10. 

Peripheral oxygen saturation, arterial blood pressure, and heart rate were recorded every 5 min after the spinal anesthesia. Any incidence of hypotension or bradycardia was reported. A > 20% decrease in mean arterial pressure, triggered immediate administration of 5 mg of ephedrine. A heart rate < 50 beats/min, triggered immediate administration of 0.5 mg of atropine.

### 3.1. Ethical Considerations

The Ethics Committee of the Shahid Beheshti University of Medical Sciences approved this study. An anesthesiologist determined the need for spinal anesthesia and explained the study's details. Patients were enrolled only after signing an informed consent. Each solution (NS, R, and HES) has been legally approved for use, and they are routinely used before spinal anesthesia for the prevention of hypotension secondary to systemic vasodilatation, and hyperbaric bupivacaine. Because the evaluation of motor and sensory block levels are routine parts of any spinal anesthesia procedure there were no additional procedures necessary.

### 3.2. Statistical Analysis

Statistical analyses were performed using SPSS software (version 17; Chicago, USA). Analysis of variance (ANOVA), chi square (or Fisher’s exact) and Kruskal-Wallis tests were used to analyze the differences between groups. The comparisons were performed using a Bonferroni correction for continuous normally distributed variables and a Wilcoxon signed rank test with a Bonferroni correction for non-normally distributed data. For measures of anesthesia we used a linear mixed model. The incidence of hypotension and bradycardia between the three groups in the clinical study was compared using a chi square test. A P value less than 0.05 was considered significant.

## 4. Results

A total of 150 patients (50 in each group) were recruited. Table 1 lists the demographic features of each group. There were no statistically significant differences in the patients; mean age, weight, height, BMI, or the number of males (Table 1). Position, lack of spinal cord deformity, bevel direction, injection speed and temperature of injected solution were similar in the three groups (P = 0.6). The median sensory nerve block level of the NS preload group (T7.36 ± 1.7) was higher than that of the HES and R preload groups (T8.32 ± 1.16, P=0.007, and T9.94 ± 2.15, P = 0.001, respectively). There was no difference found between the HES and R groups (P = 0.79) (Table 2). The maximum sensory block levels of the NS preload group at 15 min (T7.79 ± 1.61) were higher than those of the R preload group (T8.88 ± 2.07; P = 0.029). There were no differences between NS and HES, or R and HES (P = 0.60, P = 0.63, respectively).

The peak sensory block levels (range) were higher in the NS preload group T7 (T4–10), than those of the HES preload group T7 (T4–9) and R preload group, respectively. NS vs. R (P = 0.034); NS vs. V (P = 0.010)

**Figure 1.**
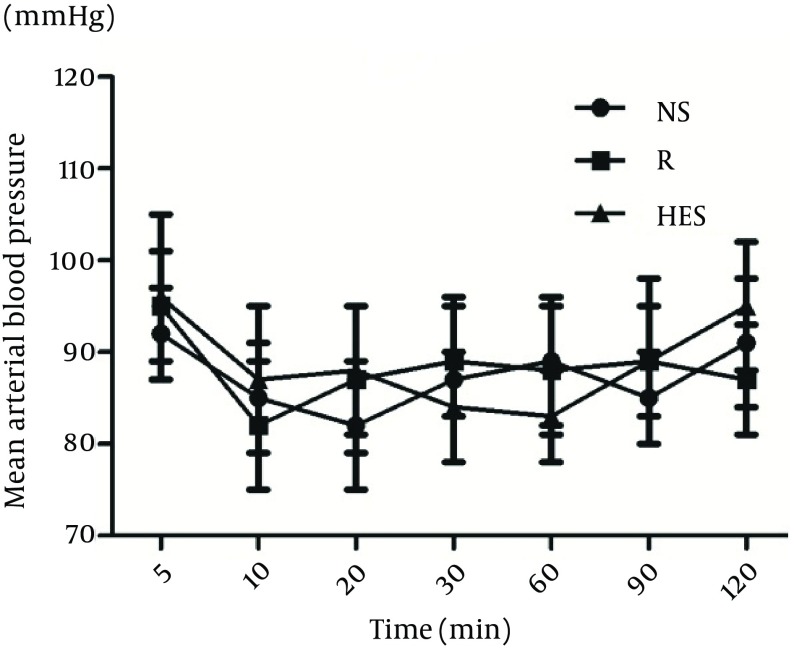
Mean Arterial Blood Pressure in Three Groups

**Figure 2.**
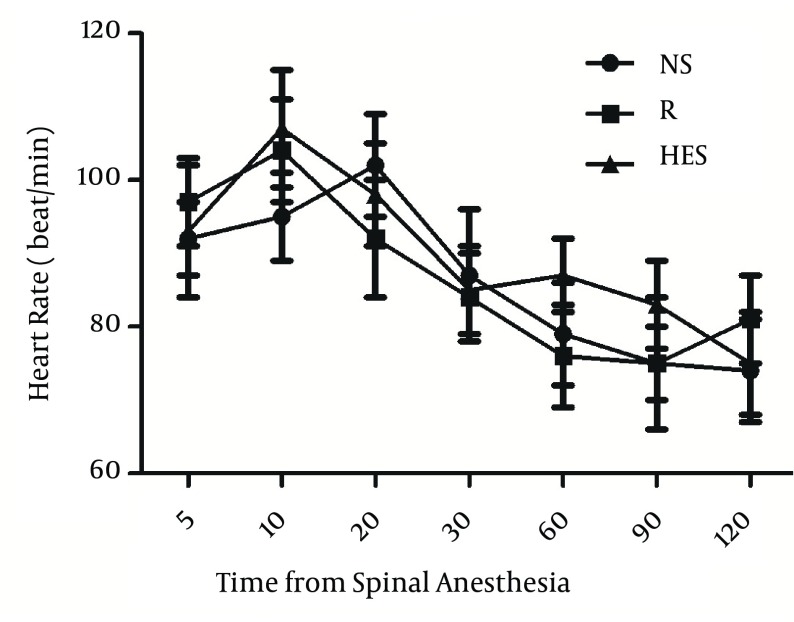
Heart Rate in Three Goups

More time was necessary to reach T10 sensory block in the R group (13.6 ± 2.3 min) than in the NS and HES groups (6.9 ± 1.3 min, P < 0.001, and 8.3 ± 1.6 min, P = 0.003, respectively); Table 2. The duration of sensory block level T10 was longer in the NS group (117.3 ± 8.6 min) compared with the HES and R groups (92.4 ± 10.2 min, P < 0.0001 and 98.4 ± 9.3 min, P = 0.002, respectively); Table 2. There were no significant differences detected between the HES and R preload groups. The time to two-dermatome regression from peak sensory block level and duration of surgery were similar in the three groups. There were no differences in mean arterial blood pressure and heart rate among the three groups at any time throughout the study (Figure 1, 2). No patient was transfused due to hemorrhage. In addition, the incidence of nausea and vomiting were also similar in the three groups (Table 3).

**Table 1.** Characteristics of Patients in the Three Preload Fluids Groups ^a^

-	Normal Saline	Ringer	Hydroxyethyl Starch	P Value
**Age, y**	35.2 ± 7.3	38.3 ± 9.4	36.9 ± 7.7	0.19
**Sex (male/female)**	39/11	41/9	42/8	0.28
**Height, cm**	169.3 ± 15.5	171.5 ± 12.2	173.1 ± 14.2	0.32
**Body weight, kg**	71.3 ± 9.3	68.9 ± 11.3	72.5 ± 10.4	0.57
**Body mass index, kg/m2**	24.6 ± 4.7	25.3 ± 3.2	23.5 ± 4.6	0.61
**ASA l/ll**	44/6	42/8	41/9	0.44

^a^ Values are presented as mean ± SD.

**Table 2.** Comparison of Spinal Anesthesia Characteristics ^a,b^

-	Normal Saline	Ringer	Hydroxyethyl Starch	P Value
**Median nerve block (spinal levels)**	T7.36 ± 1.7	T 9.94 ± 2.15	T 8.32 ± 1.16	< 0.05 ^c^
**Maximum block (spinal levels)**	T 7.79 ± 1.61	T 8.88 ± 2.07	T 7.29 ± 1.1	< 0.05 ^d^
**Peak sensory block level, range**	T7 (T4-T9)	T9 (T6-T12)	T7 (T4-T10)	< 0.05 ^e^
**Time to T10 sensory block level, min**	6.9 ± 1.3	13.6 ± 2.3	8.3 ± 1.6	< 0.05 ^f^
**Duration of sensory block >T10, min**	117.3 ± 8.6	98.4 ± 9.3	92.4 ± 10.2	< 0.05 ^g^
**Time to regression by 2 dermatomes, min**	67.4 ± 12.3	68.3 ± 9.5	62.5 ± 10.15	0.5
**Duration of surgery, min**	76.4 ± 21.2	78.1 ± 17.4	81.3 ± 22.1	0.44

^a^ Values are mean ± standard deviation, median (interquartile range), or No. (%).

^b^ Comparisons are made by ANOVA with Bonferoni and Kruskal-Wallis.

^c^ NS vs. RL (P = 0.001); NS vs. HES (P = 0.007).

^d^ NS vs. RL (P = 0.029).

^e^ NS vs. R (P = 0.034); NS vs. V (P = 0.010).

^f^ RL vs. NS (P = 0.001); RL vs. HES (P = 0.003).

^g^ NS vs. RL (P = 0.001); NS vs. HES (P = 0.002).

**Table 3.** Frequency of Adverse Effects ^a^

-	Normal Saline	Ringer’s Lactate	Hydroxyethyl Startch	P Value
**Hypotension **	15 (30)	13 (26)	13 (26)	0.76
**Bradycardia**	9 (18)	8 (16)	7 (14)	0.78
**Incidence of nausea and vomiting**	19 (38)	22 (44)	20 (40)	0.69

^a^ Data are presented as No. (%).

## 5. Discussion

In our study, the choice of preload solution before spinal anesthesia with hyperbaric bupivacaine impacted median sensory block, maximum block, peak sensory block level, time to reach to T10 and duration of sensory block at T10. Patients receiving NS showed a significantly shorter mean time to reach the peak sensory block and a longer median sensory block at 15 minutes than those receiving HES and RL. The duration of the sensory block was longest in the NS group. In a clinical study of isobaric spinal anesthesia with tetracaine, Shin et al. demonstrated that patients who received RL took a longer time to reach peak sensory block, and had a lower median sensory block at 15 and 20 minutes, than those receiving hydroxyethyl starch (12). This finding is consistent with this present study.

Crystalloid administration may dilute the CSF. This is unlikely to be a problem if the block is formed quickly. Physical and chemical differences in CSF after preload may be caused by differences in the quality of the blocks. Ion exchange in CSF is known to be caused by both static and dynamic changes in the CSF (9, 12, 13). Therefore, based on the results of our study, in addition to physical changes, the administration of crystalloid or colloid is more likely to prescribe changes in the chemical properties of CSF, like sodium levels in the CSF, as well as the intra-cellular dependent sodium channels. More time was required to reach peak sensory block level in the Rpreload group, than in the NS and HES preload groups. This needs to be taken into account when planning surgery.

Shin et al. studied the effect of Ringer’s lactate versus colloid solution before performing spinal anesthesia with isobar tetracaine. The time needed to reach maximum sensory block was greatest in the Ringer’s lactate solution group (12). They compared the changes in CSF volume and flow, time needed for maximum sensory block and median sensory block, after the administration of crystalloid solution (Ringer’s lactate 15 cc/kg) with Hextend (hetastarch containing electrolytes, 5 cc/kg). The rapid administration of large amounts of crystalloid before spinal anesthesia caused changes in CSF flow and vibrational movement, leading to changes in the distribution pattern of intra-spinal anesthetic. Time to reach maximum sensory block in the Ringer’s lactate group was delayed in comparison to the hetastarch group. The median sensory block at the 15th minute and 20th minute was lower than in the hetastarch group (9).

In contrast with previous studies which showed that the incidence of hypotension, and associated nausea and vomiting, were significantly lower in the colloid than in the crystalloid groups (14, 15), our results showed that the incidence of hypotension, bradycardia, and associated nausea and vomiting, were not significantly different in the colloid or the crystalloid groups. 

This study had several limitations. For practical reasons related to access to magnetic resonance imaging (MRI), it was not possible to examine the pulsatile movements of CSF at the L2-3 intervertebral space and midportion of the aqueduct of Sylvius. The position of the patient and baricity of the solution are believed to be the most important determinants of the spread of spinal anaesthesia (4). However, it has been reported that posture does not control the spread of a hyperbaric solution as much as was once thought (16). Injection speed was controlled, but it is difficult to strictly control it in this kind of study. On the other hand, it has been reported that different speeds of administration of hyperbaric bupivacaine do not induce any changes in anesthesia level (17). Therefore, these two factors probably had no effect on the results of this study. Other factors which may affect the spread of anesthesia, including; age, height, and BMI, were not taken into account (4). We note that in our country, using spinal anesthesia with hyperbaric solution; age, body weight, height, and gender, were not predictive factors of spinal anesthesia level. This may be because the spread of anaesthesia is dependent upon factors (such as CSF volume) which cannot be accurately predicted (based upon anthropomorphic measurements) without a MRI scan and sophisticated calculations (4).

In conclusion, we observed that NS preload had the most effect on the spread of hyperbaric bupivacaine anesthesia by higher median and maximum sensory block levels and required a shorter time to reach the peak sensory block level in comparison with the HES and R groups. Furthermore, the duration of sensory block level was the longest in the NS group.
